# Evaluation of the Effect of COVID-19 Vaccination on Exacerbations of Chronic Obstructive Pulmonary Disease: A Single-Center Study

**DOI:** 10.7759/cureus.32751

**Published:** 2022-12-20

**Authors:** Tugce Sahin Ozdemirel, Esma Zenbilli, Kerem Ensarioglu, Derya Hosgun, Busra Balkay Babaev, Melike Ak Ayaroglu, Caglar Ertugrul, Berna Akıncı Özyürek

**Affiliations:** 1 Pulmonary Medicine, Ankara Atatürk Sanatorium Training and Research Hospital, Ankara, TUR; 2 Pulmonology, Ankara Atatürk Sanatorium Training and Research Hospital, Ankara, TUR; 3 Critical Care Medicine, Ankara Atatürk Sanatorium Training and Research Hospital, Ankara, TUR; 4 Pulmonary Medicine, Atatürk Chest Diseases and Thoracic Surgery Training and Research Hospital, Ankara, TUR

**Keywords:** coronavirus disease 2019, chronic obstructive pulmonary disease, pneumococcal vaccine, influenza, vaccines, covid-19, exacerbation, copd

## Abstract

Background and aim: Vaccinations have been one of the main approaches to reducing mortality and exacerbations caused by infectious agents in chronic obstructive pulmonary disease (COPD). Among viral pathogens, coronaviruses have been described to play a role. This study aims to investigate the role of coronavirus disease 2019 (COVID-19) vaccination on exacerbation reduction in patients with COPD.

Methods: Patients diagnosed with COPD prior to the study date were considered the study population. Exacerbations of COPD before and after the COVID-19 vaccination were recorded. Patients with influenza and/or pneumococcal vaccination were excluded from the study due to their known role in reducing exacerbations of COPD.

Results: The study included 152 patients with a mean age of 67.5 ± 9.7 years. Most patients were classified under Global Initiative for Chronic Obstructive Lung Disease (GOLD) stages 2 and 3. In fully vaccinated patients, COPD exacerbation was observed to be higher than in those without full vaccination (70.5% vs. 55.3%, respectively). Total risk status and vaccination status, however, were seen to be in a positive correlation, with higher risk and complete vaccination status presenting with a higher count of COPD exacerbation.

Conclusion: Although it is known that the administration of COVID-19 vaccines in patients in risk groups reduces the risk of disease, there is no study showing a positive effect on COPD exacerbations alone. In our study, it was observed that only the COVID-19 vaccine was ineffective in attacks without influenza and pneumococcal vaccines.

## Introduction

Chronic obstructive pulmonary disease (COPD) is a respiratory disease that is inflammatory and mostly related to chronic exposure to gases and particles, mainly cigarette smoking, characterized by being partially reversible and progressively increasing airway obstruction. A COPD exacerbation is defined as a medical condition that warrants additional treatment changes in a patient's standard COPD regimen, along with increased dyspnea, coughing, and an increase in sputum production or purulence [1-3].

COPD is known to be related to an overall increase in airway sensitivity to respiratory infections [1,2]. For infection-related COPD exacerbations, 40-50% of them are caused by bacterial infections, with the remaining 30% being viral, 10% related to atypical agents, and the remaining 10-20% having multiple infectious pathogens [1]. Many viruses, mainly rhinoviruses, followed by influenza, parainfluenza, and now more commonly seen coronaviruses, are considered the viral causes of COPD exacerbations [3,4]. Newly defined severe acute respiratory syndrome coronavirus 2 (SARS-CoV-2) is responsible for coronavirus disease 2019 (COVID-19), and, as expected, the pandemic caused by it affects patients with COPD severely [5].

One of the pharmacological approaches to reduce the risk of exacerbation in COPD is vaccines [6]. Current guidelines recommend influenza and pneumococcal vaccination for patients with COPD because of the prevention of exacerbations [7]. Influenza vaccination has been demonstrated to have a protective role in patients with COPD [8,9]. For COVID-19 infection, vaccinations have also been proven to be safe and effective [10-12]. The role of COVID-19 vaccination on COPD exacerbations remains an issue to be clarified, especially considering the positive effects of other viral vaccinations and their roles on COPD. In our study, we aimed to investigate the relationship between the COVID-19 vaccine and the frequency of acute exacerbations in patients with COPD.

## Materials and methods

Study design

The study was performed as a single-center retrospective cohort in a tertiary care hospital specialized in pulmonary medicine and approved by the local ethics committee (date: February 17, 2022; number: 24). Written informed consent was obtained from all participants who participated in this study. Patients diagnosed with COPD prior to the study date with at least one COPD-related routine chest disease outpatient clinic evaluation were considered as the study population. Patients with influenza in the last one year and/or pneumococcal vaccination in the last five years were excluded from the study due to their known role in reducing COPD exacerbations.

The patients who applied to our chest disease outpatient clinic were questioned about their emergency service admissions, the number and duration of hospitalizations, and their history of hospitalization in the intensive care unit before and after the COVID-19 vaccination. The demographic characteristics of the patients and their COVID-19 vaccination histories were recorded.

COPD exacerbation was considered the study's primary outcome. Survival, hospitalization history, and duration were accepted as secondary outcomes. Regarding the confounding parameters, age, gender, and the presence of additional comorbidities were considered the main confounding parameters that could have affected exacerbation and hospitalization requirements. Those who had three vaccines (two Sinovac + one BioNTech or two BioNTech or three Sinovac) were considered fully vaccinated according to the COVID-19 vaccination program of the Ministry of Health of the Republic of Turkey. Risk stratification was utilized for defining high-risk groups, in which those with more than two emergency service visits or with at least one COPD-related hospital admission were defined as high-risk patients [13].

Data collection

Data were collected through a search of the hospital's digital information system. Demographic characteristics (including patients' age and sex), laboratory parameters at admission (including complete blood count, liver and renal functional test, C-reactive protein (CRP), and D-dimer levels), pulmonary functional data, and COVID-19 vaccination status were recorded.

Statistical analysis

As per the study's retrospective design, no loss to follow-up was expected; thus, no specific analysis was planned. In the case of outliers for unexpectedly high COPD exacerbations (more than 12/year), an analysis without outliers was planned. Regression analyses were planned to investigate the role of parameters regarding COPD exacerbation. IBM SPSS version 25 (IBM Corp, Armonk, NY) was used as the statistical analysis program.

## Results

The study included 152 patients (32 females/120 males) with a mean age of 67.5 ± 9.7 years. Most of the patients (n = 114, 75%) had at least one additional known comorbidity. The average number of COVID-19 vaccination was found to be 2.9 (±0.93), in favor of Sinovac (1.94 ± 1.19 vs. 1.23 ± 1.05), and 69.1% (n = 105) of the patients were accepted as fully vaccinated (Tables 1, 2).

**Table 1:** Demographic characteristics ^1^ A total of three COVID-19 vaccinations, regardless of the combination, and the last dosage being done at least 28 days earlier than the initial evaluation was defined as "full vaccination." ^2 ^Polyclinic and emergency admissions were evaluated after the last known history of vaccination (if a patient had at least one vaccination) or from the initial polyclinic evaluation if a patient had not been vaccinated. ^3^ Any COVID-19 reverse-transcription polymerase chain reaction (RT-PCR) positivity during the follow-up was accepted as a positive result for COVID-19 infection. All parameters are given with either percentage or with one standard deviation.

	Fully vaccinated^1^ (N = 105, 69.1%)	Incomplete or no vaccination^1^ (N = 47, 30.9%)	Total (N = 152)
Age (years)	69 (±9.2)	64 (±9.9)	67.5 (±9.7)
Gender			
Male	89 (84.8%)	31 (66%)	120 (78.9%)
Female	16 (15.2%)	16 (34%)	32 (21.1%)
Polyclinic admission^2^	2.64 (±2.39)	2.51 (±2.03)	2.62 (±2.2)
Emergency admission^2^	1.44 (±2.1)	2.64 (±4.42)	1.8 ((±3.0)
COVID-19 infection^3^	33 (31.4%)	14 (29.8%)	47 (30.7%)
ICU admission	4 (3.8%)	3 (6.4%)	7 (4.6%)
One-year survival	103 (99%)	46 (97.9%)	140 ((97.4%)
Vaccination for COPD exacerbation (days)	88.56 (±62.6)	93.34 (±88.5)	90 (±70.9)
Average hospitalization duration (days)	4.64 (±7.42)	4.09 (±5.36)	4.47 (±6.84)

**Table 2:** Comorbidity comparison ^1^ A total of three COVID-19 vaccinations, regardless of the combination, and the last dosage being done at least 28 days earlier than the initial evaluation was defined as "full vaccination."

	Fully vaccinated^1^ (N = 105, 69.1%)	Incomplete or no vaccination^1^ (N = 47, 30.9%)	Total (N = 152)
Additional comorbidity	79 (75.2%)	35 (75%)	114 (75%)
Diabetes mellitus	25 (23.8%)	11 (23.4%)	36 (23.6%)
Cardiac disease history	34 (32.4%)	19 (40.4%)	53 (34.8%)
Hypertension	53 (50.5%)	14 (29.8%)	67 (44%)
Pulmonary disease history	19 (18.1%)	4 (8.5%)	23(15.1%)
Immunosuppression presence	11 (10.5%)	2 (4.3%)	13 (8.6%)
Neurologic disease history	9 (%8.6)	6 (12.8%)	15 (9.8%)
Other comorbidities	17 (16.2%)	7 (14.9%)	24 (15.7%)

Polyclinic admission after vaccination was found to be 2.6 (±2.2) counts, while emergency admission was observed to be 1.8 (±3.0) in total. Intensive care unit (ICU) admission was observed only in 4.6% (n = 7) of all patients, and the overall one-year survival was 97.4% (n = 149), stating that most patients survived the initial follow-up period. The average duration between the last COVID-19 vaccination to the following COPD exacerbation was 90 days (29-131), and the average hospitalization duration was four days (0-8) (Table 1).

The COPD exacerbations before vaccination completion were observed to be one (0-17) attack yearly, with the exacerbation rate after vaccination at two (0-30) attacks yearly. COPD exacerbations in absolute value were seen in 65.4% (n = 100) of patients and high-risk patients had a higher COPD exacerbation count and ratio compared to the low-risk patients (Table 3).

**Table 3:** Vaccination, COPD exacerbation, and hospital admissions ^1 ^A total of three COVID-19 vaccinations, regardless of the combination, and with the last dosage being done at least 28 days earlier than the initial evaluation was defined as "full vaccination." ^2 ^Risk status was evaluated by dividing patients into two categories according to the GOLD classification, with groups A and B being “low risk” and groups C and D defined as “high risk.” All parameters are given with either percentage or with one standard deviation. COPD: chronic obstructive pulmonary disease; GOLD: Global Initiative for Chronic Obstructive Lung Disease.

Vaccination status^1^	Fully vaccinated			Incomplete or no vaccination			Total (N = 152)
Risk status^2^	Total (N = 105)	High risk (N = 59)	Low risk (N = 46)	Total (N = 47)	High risk (N = 28)	Low risk (N = 19)	
COPD exacerbation before COVID-19 vaccination	1 (0-17)	1 (0-17)	1 (0-7)	1 (0-15)	1.5 (0-15)	0 (0-3)	1 (0-17)
COPD exacerbation after COVID-19 vaccination	2 (0-19)	2.5 (0-19)	2 (0-6)	2 (0-19)	3 (0-30)	1 (0-4)	2 (0-30)
Increase in absolute COPD exacerbation count	74 (70.5%)	44 (74.5%)	30 (65.2%)	26 (55.3%)	19 (67.8%)	7 (36.8%)	100 (65.4%)

In fully vaccinated patients, COPD exacerbation count was observed to be higher than those without full vaccination (70.5% vs. 55.3%, respectively). The risk and odds ratios for those with vaccination to have higher COPD exacerbation numbers were 1.55 (95% CI: 0.97-2.47) and 1.92 (95% CI: 0.94-3.92). The statistical correlation with Pearson's chi-square between increased COPD exacerbation and vaccination was not found significant (p = 0.095; Table 4).

**Table 4:** Risk evaluation of vaccination in COPD exacerbations ^1^ A total of three COVID-19 vaccination, regardless of the combination, and with the last dosage being done at least 28 days earlier than the initial evaluation was defined as "full vaccination.” ^2^ The given p-value is estimated from Pearson's chi-square results and is two-sided. COPD: chronic obstructive pulmonary disease; RR: risk ratio; OR: odds ratio; CI: confidence interval.

	Fully vaccinated^1 ^(N = 105)	Incomplete or no vaccination^1 ^(N = 47)	Total (N = 152)	95% CI, lower	95% CI, upper	P-value^2^
Increase in absolute COPD exacerbation count	74 (70.5%)	26 (55.3%)	100 (65.7%)			0.095
No increase in absolute COPD exacerbation count	31 (29.5%)	21 (44.7%)	52 (34.3%)			
RR			1.55	0.97	2.47	
OR			1.92	0.94	3.92	

Binomial regression analysis was performed for the evaluation of risk status, vaccination status, gender, and age as parameters for COPD exacerbation. Gender was found not to be a significant parameter, while age, risk status, and vaccination status were observed to be significant parameters (p = 0.014, 0.006, and 0.012, respectively). The patients’ age was calculated to be a negative parameter in terms of exacerbation, with elderly patients having more exacerbations. Total risk status and vaccination status, however, were seen to be in a positive correlation, with higher risk and complete vaccination status presenting a higher COPD exacerbation count (Table 5).

**Table 5:** Binomial regression analysis between absolute reduction in COPD exacerbations and other parameters ^a ^0 = low risk; 1 = high risk. ^b^ 0 = incomplete or no vaccination; 1 = fully vaccinated. SE: standard error; CI: confidence interval.

	B	SE	Wald	Exp(B)	95% CI for Exp(B)		p
					Lower	Upper	
Total risk^a^	1.025	0.373	7.534	2.43 (1.21-4.89)	1.340	5.791	0.006
Vaccination status^b^	1.054	0.419	6.333	2.19 (1.00-4.76)	1.263	6.525	0.012
Gender	0.299	0.468	0.408	0.79 (0.38-1.67)	0.539	3.375	0.523
Age	-0.051	0.021	6.053	0.79 (0.38-1.67)	0.913	0.990	0.014
Constant	2.786	1.345	4.292	0.80			0.038

## Discussion

In this study, we investigated whether there was a decrease in hospital admissions due to an exacerbation in COPD patients after COVID-19 vaccination. It was determined that there was no decrease in the frequency of COPD exacerbations, even an increase, within one year after the administration of the COVID-19 vaccine in COPD patients when compared to the previous year.

The most important risk factor for COPD exacerbation is a history of exacerbation in the previous year [14,15], and in the literature, there is a significant correlation between the stages of COPD and the frequency of exacerbations [16]. In our study, most patients were under the Global Initiative for Chronic Obstructive Lung Disease (GOLD) grades 2 to 3 and were at higher risk for exacerbations [13]. The weak yet positive correlation between former COPD exacerbation history and exacerbation after COVID-19 vaccination was an expected result due to the former COPD exacerbation history itself being one of the most prominent risk factors for repeated exacerbations. The additional increase over the follow-up period was assumed to be present, as per the progressive nature of COPD; however, a statistically significant increase was not expected. Such an increase might have masked the effect of other parameters regarding COPD control, in this case, the vaccination's possible protective role.

The additional comorbidities of the patients were observed to be present in the majority of the study group, which, combined with the advanced age of the patients, supported the initial assumption of the group being considered fragile compared to the general population. In accordance with the literature, these factors effectively played a role in high COPD exacerbations in our study [17,18]. There are many studies in the literature stating that there is a negative correlation between forced expiratory volume in one second (FEV1) levels and hospital stay [19,20]. In our study, overall survival was found to be high, and ICU admission was observed to be low; thus, it can be stated that, albeit fragile, the patient group had yet to reach the terminal stage of COPD. When evaluated together with fragility, polyclinic, and emergency admissions, we can categorize most patients under GOLD grades 2 to 3 [13].

As of writing this study, there were no similar studies that evaluated the role of COVID-19 vaccination for COPD exacerbation. It has been reported in the literature that influenza and pneumococcal vaccination significantly reduces exacerbations of COPD [8,9]. In our study, we wanted to investigate the effect of COVID-19 vaccination by excluding those with a history of these two vaccinations. According to our findings, we concluded that influenza and/or pneumococcal vaccination is more effective in reducing exacerbations in COPD exacerbations, whereas COVID-19 vaccination may be insufficient in this regard [21]. Coronavirus has been found to increase exacerbation in patients with COPD [22]. The effectiveness of the COVID-19 vaccine or its effectiveness against infection and symptomatic disease decreases by approximately 20-30% in six months [22]. We investigated the exacerbations of COPD for one year after COVID-19 vaccination. Additionally, because we are in a pandemic, patients may have been diagnosed with an unconfirmed COVID-19, which has been termed an exacerbation of COPD. Symptoms that occur due to the prolonged effect of immune reactions after COVID-19 vaccination may be perceived as an exacerbation of COPD.

It has been stated in the literature that age is a risk factor for the exacerbation of COPD [15]. In our study, the fact that the number of exacerbations was relatively higher in those who were fully COVID-19 vaccinated compared to those who were not vaccinated can be explained by this situation. In addition, the higher prevalence of hypertension in full-vaccinated patients may be associated with increased exacerbation. Although the relationship between COPD and hypertension is not fully known yet, it is suggested that acceleration of aging, loss of connective tissue, and an increase in “arterial stiffness” may play a role in the emergence of hypertension [23].

The results remain limited in generalizability due to the retrospective nature of the study and the exact cut-off of vaccination being a variable parameter among different countries and centers. As stated earlier, we assume that studies made within the same patient population as ours would lead to a similar result of statistical significance or barely to the point that, in multifactorial analyses, the role of other parameters may overshadow the vaccination's role.

The limitations of our study were that it was retrospective, it was not evaluated according to the stage of the patients with COPD, and the number of patients was low.

## Conclusions

The reducing effect of both pneumococcal and flu vaccines on exacerbations of COPD has been shown in the literature. Although it is known that the administration of the COVID-19 vaccine in patients in risk groups reduces the risk of disease, there is no study showing a positive effect on COPD exacerbation alone. In our study, it was observed that the COVID-19 vaccine was ineffective on exacerbations without influenza and pneumococcal vaccines. Further studies are required for the evaluation of the role of COVID-19 vaccinations in other COPD patient groups.
